# *GATA1* epigenetic deregulation contributes to the development of AML with *NPM1* and *FLT3-*ITD cooperating mutations

**DOI:** 10.1038/s41375-019-0399-7

**Published:** 2019-02-12

**Authors:** Paolo Sportoletti, Letizia Celani, Emanuela Varasano, Roberta Rossi, Daniele Sorcini, Chiara Rompietti, Francesca Strozzini, Beatrice Del Papa, Valerio Guarente, Giulio Spinozzi, Debora Cecchini, Oxana Bereshchenko, Torsten Haferlach, Maria Paola Martelli, Franca Falzetti, Brunangelo Falini

**Affiliations:** 1 Centro di Ricerca Emato-Oncologica (CREO), University of Perugia, Perugia, 06132 Italy; 2 Section of Pharmacology, Toxicology and Chemotherapy, University of Perugia, Perugia, 06132 Italy; 3 MLL Munich Leukemia Laboratory, Munich, 81377 Germany

**Keywords:** Acute myeloid leukaemia, Diseases, Haematological diseases

## To the Editor:

About 20 recurrently mutated genes are known to be involved in the molecular pathogenesis of acute myeloid leukemia (AML) [1]. Among them, *Nucleophosmin* (*NPM1*) and *FLT3*-ITD mutations frequently occur together in adults with AML [2], suggesting cooperative leukemogenesis. To date, the molecular consequences of these cooperative genetic alterations in AML are still elusive.

**Fig. 1:**
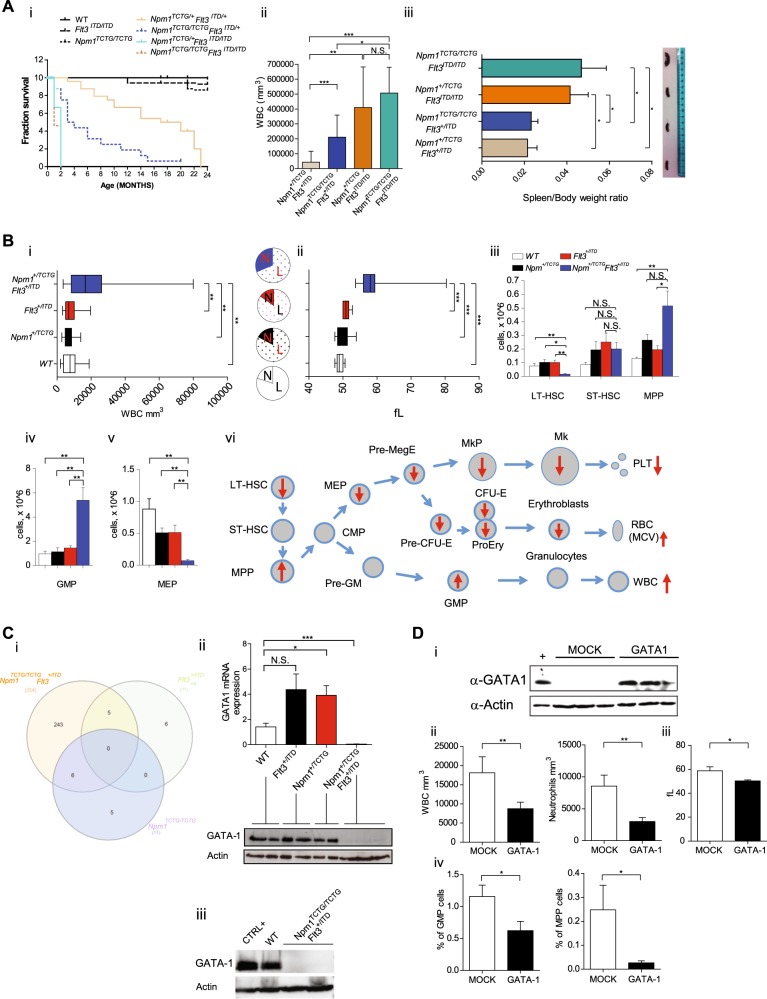
Lethal acute myeloid leukemia (AML) in *Npm1/Flt3-ITD* mice is preceded by changes in myeloid and erythroid cells associated with GATA1 deregulation. **a** (i) Kaplan−Meier plot of mouse survival according to the indicated genotypes (*n* = 8 to 24 per genotype); *Npm1*^+/TCTG^;*Flt3*^+/ITD^ mice display a median survival of 18.5 months versus 21 months of *Npm1*^+/TCTG^ or *Flt3*^+/ITD^ mice or 22.5 months of wild-type controls (*p* < 0.0001, logrank test). (ii) Changes in white blood cell (WBC) counts of *Npm1*^*+/TCTG*^*;Flt3*^*ITD/ITD*^ (*n* = 3), *Npm1*^*TCTG/TCTG*^*;Flt3*^*ITD/ITD*^ (*n* = 3), *Npm1*^*+/TCTG*^*;Flt3*^*+/ITD*^ (*n* = 6) and *Npm1*^*TCTG/TCTG*^*;Flt3*^*+/ITD*^ (*n* = 9) mice. (iii) Spleen weight to total body weight ratio in the indicated genotypes. Spleen ratio in *Npm1*^*+/TCTG*^*;Flt3*^*ITD/ITD*^ (*n* = 3) and *Npm1*^*TCTG/TCTG*^*;Flt3*^*ITD/ITD*^ (*n* = 4) mice was two fold greater than in *Npm1*^*+/TCTG*^*;Flt3*^*+/ITD*^ (*n* = 5) and *Npm1*^*TCTG/TCTG*^*;Flt3*^*+/ITD*^ (*n* = 18) leukemic mice (0.041 ± 0.014 and 0.046 ± 0.023 vs 0.017 ± 0.012 and 0.025 ± 0.016 *p* < 0.001 by one-way analysis of variance (ANOVA) analysis). **b** (i) Significant differences in WBC count in *Npm1*^*+/TCTG*^*;Flt3*^*+/ITD*^ compared to *Npm1*^*+/TCTG*^*;Flt3*^*+/+*^, *Npm1*^*+/+*^*;Flt3*^*+/ITD*^ and *Npm1*^*+/+*^*;Flt3*^*+/+*^ littermate groups (*n* = 12 to 20 per genotype); pie charts show neutrophils (N) and lymphocytes (L) percentages. (ii) Mean corpuscolar volume (MCV) values in preleukemic mice (*n* = 12 to 20 per genotype). (iii–v) Flow-cytometric analysis of bone marrow stem and progenitor cell compartment sizes, including long-term hematopoietic stem cells (LT-HSCs; lin^−^Sca-1^+^c-kit^+^ CD34^−^Flt3^−^), short-term HSCs (ST-HSCs; lin^-^Sca-1^+^c-kit^+^CD34^+^Flt3^−^), multipotent progenitors (MPPs; lin^−^Sca-1^+^c-kit^+^CD34^+^Flt3^+^), granulocyte/monocyte progenitors (GMPs Lin^−^Sca-1^−^cKit^+^CD34^+^FcγRII/III^hi^) and common megakaryocyte-erythroid progenitor (MEP; Lin^−^Sca-1^−^cKit^+^CD34^−^FcγRII/III^lo^) populations (*n* = 4 to 10 per genotype). (vi) Summary of hemopoietic changes in *Npm1*^*+/TCTG*^*;Flt3*^*+/ITD*^ mice. **c** (i) Overlap of differently gene expression profiling (GEP) of *Npm1*^*TCTG/TCTG*^*;Flt3*^*+/+*^, *Npm1*^*+/+*^*;Flt3*^*+/ITD*^ and *Npm1*^*TCTG/TCTG*^*;Flt3*^*+/ITD*^ compared to *Npm1*^*+/+*^*;Flt3*^*+/+*^ (*n* = 3 mice for each genotype). (ii) GATA1 messenger RNA (mRNA) and protein expression in the bone marrow (BM) of the indicated genotypes. (iii) GATA1 protein expression in lineage-depleted BM cells from the indicated genotypes. **d** (i) Enforced expression of GATA1 protein in the BM of mice transplanted with *Npm1*^*+/TCTG*^*;Flt3*^*+/ITD*^ LSK (*n* = 4 to 12) infected with an inducible GATA1 lentiviral system and killed 2 months after transplantation. (ii, iii) Significant differences in WBC counts, neutrophils and MCV values in the peripheral blood (PB) of GATA1-rescued mice. (iv) Frequency of MPP and GMP populations in MOCK (*n* = 10) and GATA1 (*n* = 16) infected mice. Data represent the mean ± SD. N.S. not significant; *p < 0.05, **p < 0.01, ***p < 0.001; unpaired *t*-test with Welch’s correction

To address this issue, we crossed *Npm1* and *Flt3-ITD* mutant mice demonstrating the onset of lethal AML (Fig. 1a and Figure S1). Interestingly, the cumulative mutant allele burden influenced the leukemic phenotype, penetrance and latency. *NPM1*/*Flt3*-ITD double heterozygous mice (*Npm1*^+/TCTG^;*Flt3*^+/ITD^) displayed a significantly reduced overall survival compared to single mutant or wild-type mice. Survival was further reduced in mice with two *NPM1* mutant alleles and one *Flt3*-ITD allele (*Npm1*^TCTG/TCTG^;*Flt3*^+/ITD^). Interestingly, *Flt3*-ITD homozygous mice (*Npm1*^+/TCTG^;*Flt3*^ITD/ITD^ and *Npm1*^TCTG/TCTG^;*Flt3*^ITD/ITD^) showed the higher white blood cell (WBC) counts, a significant spleen enlargement (Fig. 1a ii-iii) and developed AML rapidly regardless of *NPM1* mutation dosage, suggesting that in the context of a high FLT3 kinase activity even small NPM1 mutant levels are leukemogenic. This supports a direct oncogenic effect of the NPM1 mutant, beside the haploinsufficient tumor suppressor effects of the concomitant loss of one *NPM1* allele [3, 4]. Additionally, our data are in line with the clinical observation that normal karyotype AML with high *FLT3-ITD* levels have a poor outcome [5].

The analysis of the *Npm1*^*+/TCTG*^*;Flt3*^*+/ITD*^ genotype, which is characterized by a longer AML latency, demonstrated changes in the myeloid and erythroid cells before leukemia onset. WBC counts and mean corpuscular volume (MCV) were significantly higher in *Npm1*^*+/TCTG*^*;Flt3*^*+/ITD*^ mice than in wild-type, *Npm1*^+/TCTG^ and *Flt3*^+/ITD^ groups (Fig. 1b i-ii). Flow cytometry analysis of bone marrow (BM) populations showed that leukocytosis was associated to reduced long-term hematopoietic stem cells (HSCs), significant expansion of multipotent progenitor (MPP) cells and a 6.1-fold increase of granulocyte/monocyte progenitors (GMPs) (Fig. 1b iii-iv). *Npm1*^*+/TCTG*^*;Flt3*^*+/ITD*^ mice had decreased number of immature and recirculating B-cell BM populations (Figure S2). Erythrocyte changes reflected a significant reduction in the corresponding BM populations at different differentiation stages including myelo-erythroid progenitors (MEP), pre-megakaryocyte-erythrocyte progenitors (PreMegE), pre-colony forming unit-erythroid (pre-CFU-E), CFU-E and proerythroblasts (proEry) that resulted almost absent (Fig. 1b v-vi and Figure S3). In physiological hematopoiesis, FLT3 up-regulation is important in sustaining MPP and GMP but not MEP potential [6]. In *Npm1*^*+/TCTG*^*;Flt3*^*+/ITD*^ mice, constitutive Flt3-ITD signaling boosts the myeloid bias and influences the megakaryocyte/erythroid lineage fates, strongly suggesting the capacity of the NPM1 mutant to synergize with a FLT3 activity. These findings appear to define the cellular background for the acquisition of additional events for AML onset.

Comparative gene expression profiling (GEP) studies on total BM revealed a large number of differentially expressed genes in *Npm1*^*TCTG/TCTG*^*;Flt3*^*+/ITD*^ leukemic mice compared to *Flt3*^*+/ITD*^, *Npm1*^*TCTG/TCTG*^ and wild-type groups. A total of 254 genes were differentially expressed in *Npm1*^*TCTG/TCTG*^*;Flt3*^*+/ITD*^ mice compared to wild-type littermates (42 up-regulated; 214 down regulated) (Table S1). Interestingly, when compared with wild-type, there were 243 transcripts whose expression was changed only in *Npm1*^*TCTG/TCTG*^*;Flt3*^*+/ITD*^ cells (Fig. 1c i). There were no transcripts commonly altered in all pairwise comparisons. Hoxa9 scored as one of the most up-regulated genes in *Npm1*^*TCTG/TCTG*^*;Flt3*^*+/ITD*^ mice, a characteristic hallmark of *NPM1*-driven leukemia. Similar findings were present in mice with early-stage AML.

Pathway analysis showed different changes when comparing *Npm1*^*TCTG/TCTG*^*;Flt3*^*+/ITD*^ to wild-type mice. Among these, we found pathways involved in hematopoietic cell lineage development, the B-cell receptor signaling and the immunoregulatory interactions between lymphoid and non-lymphoid cells. Additionally, *Npm1*^*TCTG/TCTG*^*;Flt3*^*+/ITD*^ BM samples displayed a significant deregulation of factors involved in megakaryocyte development and platelet production. Interestingly, several genes associated with this pathway were linked to a GATA transcriptional signature, including GATA1, Zpfm1, Rac1 and Ehd2. The latter showed a significant downregulation, with GATA1 displaying the lower levels (Figure S4). A similar expression signature was present in lineage-depleted BM cells used to exclude biases related to the different cellular composition of leukemic versus wild-type mice (Figure S5). In this context, we found a higher number of deregulated GATA gene family members including GATA1, GATA2 and GATA3.

Results of GEPs and the presence of alterations in erythropoiesis before AML development prompted us to focus on GATA1, the master regulator of erythroid differentiation. Notably, BM changes in *Npm1*^+/TCTG^;*Flt3*^+/ITD^ mice were accompanied by a dramatic reduction of GATA1 messenger RNA (mRNA) and complete loss and substantial downregulation of protein expression in both total or lineage-depleted BM (Fig. 1c ii-iii). The extent of GATA1 deregulation correlated with the degree of the myeloid phenotypic changes (Figure S6). In vivo restoration of GATA1 expression in *Npm1*^+/TCTG^;*Flt3*^+/ITD^ Lin^−^Sca-1^+^cKit^+^ cells using a conditional lentiviral system (Fig. 1d i and Figure S7A) rescued most of the preleukemic phenotype which included a significant reduction of WBC and neutrophils in peripheral blood (PB) and a decrease in percentage of MPP and GMP (Fig. 1d ii,iv and Figure S7B). Interestingly, GATA1 re-expression also led to a rescue of macrocytosis with a significant reduction of the MCV values from 58.7 to 50.2 fL (Fig. 1d iii). Moreover, spleens from GATA1-rescued mice showed a decrease in size and a reduction of myeloid-infiltrating cells compared to controls (Figure S7C). Collectively, these findings provide evidence that deregulation of GATA1 plays a key role in the hemopoietic changes preceding AML in *Npm1*^+/TCTG^;*Flt3*^+/ITD^ mice. This is consistent with the concept that in blood cell precursors, GATA1 is necessary for erythroid lineage differentiation and antagonizes the activity of myeloid transcription factors [7].

Our findings are consistent with the observation that *GATA1* heterozygous knock-out female mice frequently develop a myeloproliferative disorder with a splenic accumulation of proerythroblasts and megakaryocytes, anemia and thrombocytopenia [7]. Moreover, recurrent GATA1 mutations abrogating the expression of the full-length GATA1 have been found in myeloid proliferations related to Down syndrome, including transient abnormal myelopoiesis and megakaryoblastic AMLs [8]. Interestingly, *FLT3*-ITD mutations were more frequent in AML patients who lacked GATA1 expression [9] and even *IDH*-mutated AML patients displayed a distinct methylation signature, including the aberrant hypermethylation of GATA1/2 gene promoter [10].

**Fig. 2:**
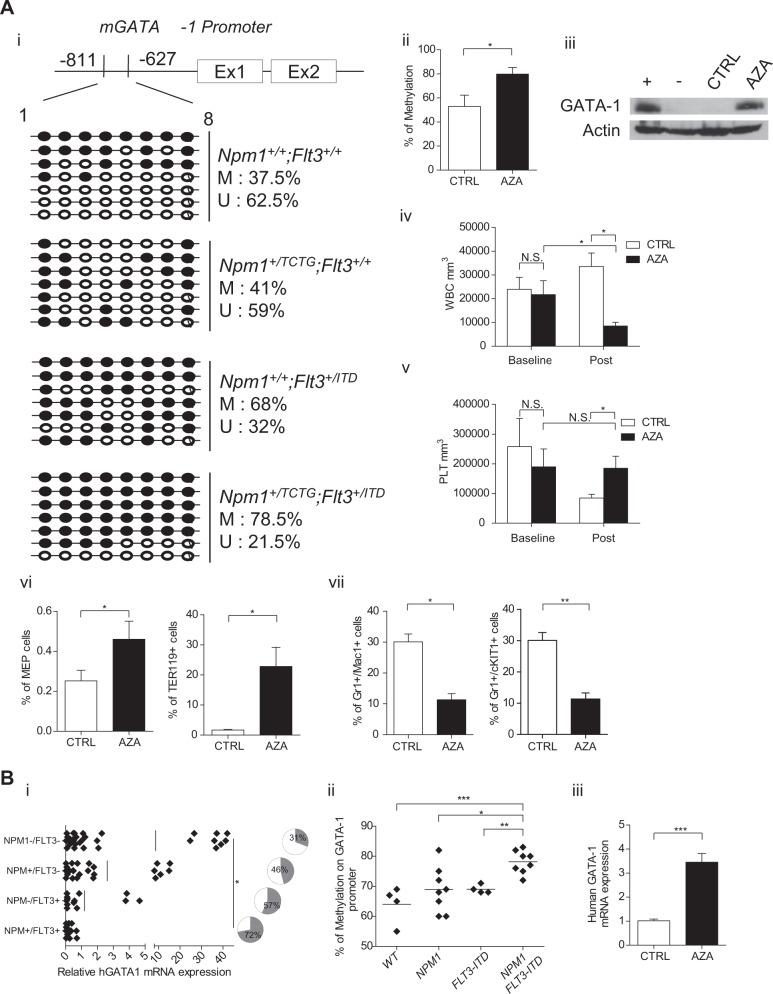
Decreased GATA1 expression levels in human and mouse *NPM1*/*FLT3-ITD* mutated acute myeloid leukemia (AML) depends on promoter methylation. **a** (i) Analysis of DNA methylation at the mouse *GATA1* locus by sequencing of PCR clones derived from sodium bisulfite–treated mouse genomic DNA extracted from the bone marrow (BM). Each row of circles represents the sequence of an individual clone; open circles indicate unmethylated CpG sites and closed circles indicate methylated CpG sites. (ii) Methylation status of the GATA1 promoter as determined by the Methylight assay. (iii) GATA1 protein expression in the BM of Aza-treated mice (*n* = 5). (iv, v) Changes in white blood cell (WBC) and platelet (PLT) counts of *Npm1*^*+/TCTG*^*;Flt3*^*+/ITD*^ mice treated with Aza (*n* = 5 to 10 per treatment group). (vi) Frequencies of MEP and Ter119 cells in the BM of Aza-treated mice (*n* = 5 to 12 per treatment group). (vii) Frequencies of Gr1+/cKit+ immature and Gr1+Mac1+ mature myeloid cells in the spleen of untreated vs 5-Aza-treated leukemic mice (*n* = 5). **b** (i) GATA1 mRNA average expression in AML patients with NPM1/FLT3-ITD mutation compared to unmutated, NPM1 and FLT3-ITD single mutant (*p* < 0.05 comparing all the groups); pie charts indicate the percentage of patients with GATA1 expression below the median in the indicated mutation group. (ii) GATA1 promoter methylation frequency in AML patients with NPM1/FLT3-ITD mutation (*n* = 8) as compared to unmutated (*n* = 4), NPM1 (*n* = 8) and FLT3-ITD (*n* = 4) single mutant. (iii) GATA1 mRNA levels in the BM of human AML patients (*n* = 3) before and after in vivo Aza treatment. N.S. not significant; *p < 0.05, **p < 0.01; ***p < 0.001; unpaired *t*-test with Welch’s correction

Proteasome inhibition of *Npm1*^+/TCTG^;*Flt3*^+/ITD^ BM cells in vitro did not rescue GATA1 protein expression (Figure S8A), suggesting that *NPM1* and *Flt3*-ITD mutations regulate GATA1 transcription. Thus, we explored changes in the methylation status of the GATA1 promoter region (from −811 to −627 bp) and observed dense DNA methylation in *Npm1*^+/TCTG^;*Flt3*^+/ITD^ samples (Fig. 2a i-ii). To support GATA1 epigenetic silencing as a mechanism favoring AML, we treated *Npm1*^+/TCTG^;*Flt3*^+/ITD^ mice with the DNA methyltransferase inhibitor 5-aza-deoxycytidine (5-Aza-dC). This resulted in the reactivation of GATA1 expression in BM (Fig. 2a iii and Figure S8B), normalization of leukocytosis and prevention of a drop in platelet counts (Fig. 2a iv-v). Although 5-Aza-dC treatment had no impact on MCV, both MEP and Ter119 cells were significantly expanded in treated animals (Fig. 2a vi). Flow cytometry of spleen demonstrated a significant reduction of both mature and immature myeloid cells in 5-Aza-dC-treated mice (Fig. 2a vii). Our findings are reminiscent of the differential methylation of GATA target genes previously reported in AML mouse models combining *FLT3*-ITD to either IDH mutants [11] or *TET2* loss [12]. The higher 5’-GATA1 methylation in *Npm1*^*+/TCTG*^*;Flt3*^*+/ITD*^ mice points to a gene dose effect for GATA1 during leukemogenesis being finely tuned by CpG methylation. This suggests that *NPM1* alterations may contribute to epigenetic modifications, especially in the presence of other mutations, such as *Flt3-*ITD. This view is consistent with NPM1 being an histone chaperone that interacts with linker histone H1, plays a role in sperm chromatin remodeling, enhances acetylation-dependent chromatin transcription and controls ribosomal DNA gene transcription [13].

To assess the relevance of mouse findings to human AML, we correlated GATA1 mRNA expression with the *NPM1* and *FLT3*-ITD mutational status in the BM of 47 AML, demonstrating that patients harboring both mutations displayed the lowest expression of GATA1 (Fig. 2b i). The median GATA1 level of 0.44 was arbitrarily used as cut-off to distinguish high and low expressing patients. AMLs with low GATA1 were more frequent among *NPM1*-mutated/*FLT3-ITD* AMLs than unmutated, single *NPM1* or *FLT3*-ITD-mutated patients (72 vs 31, 46 and 57% respectively; pie charts in Fig. 2b i). These findings were further validated in an independent database of 266 AML of the Munich Leukemia Laboratory (www.ncbi.nlm.nih.gov/geo, accession number (GSE16015) (Figure S9). Additionally, we explored the methylation status of the GATA1 promoter region in 24 patients, revealing a significant DNA methylation in *NPM1*-mutated/*FLT3-ITD* samples with an average of 78.1% ± 1.3 methylated CpG sites compared to 68.8% ± 2.6 in *NPM1*-mutated only, 69% ± 0.7 in *FLT3*-ITD-mutated only and 64% ± 3.1 in wild-type (Fig. 2b ii). Finally, the analysis of GATA1 expression levels was performed in BM samples from 3 *NPM1*-mutated/*FLT3-ITD* patients treated with 5-Aza-dC, revealing a significant up-regulation of GATA1 mRNA after the first cycle (Fig. 2b iii). These data corroborate a potential role for DNA methylation of GATA1 promoter in the development of *NPM1*-mutated/*FLT3-ITD* AML. Our findings are also of potential clinical relevance, as GATA1 transcriptional response to 5-Aza-dC in mice results in significant improvement of the myeloid phenotype. Similarly, we observed GATA1 mRNA up-regulation in two *NPM1*-mutated/*FLT3-ITD* AML patients upon 5-Aza-dC treatment.

In conclusion, we identified deregulation of GATA1 as a new feature of *Npm1*/*Flt3*-ITD AML in mice and humans. This is an early event altering the HSC fate and sensitizing cells to further malignant transformation. Our model may also be valuable for further assessment of FLT3 inhibitors [14] and other drugs that have been shown to be active against *NPM1*-mutated AML [15].

## Supplementary information

Supplementary Material
