# Supplementary material for: GATA1 epigenetic deregulation contributes to the development of AML with NPM1 and FLT3-ITD cooperating mutations
Source: Leukemia. 2019 Feb 12;33(7):1827–32. doi: 10.1038/s41375-019-0399-7 (PMC6756200; doi:10.1038/s41375-019-0399-7)
Supplement: Supplementary file 1 — Supplementary Material [file 41375_2019_399_MOESM1_ESM.pdf]

MATERIAL & METHODS

Mouse strains and treatments

Mice were housed by the "Service center of Preclinical Research" of Perugia and manipulations were performed according to the protocol approved by the Italian Health Ministry. To generate compound *Npm1*/*Flt3*-ITD mutant animals, we crossed conditional *NPM1*[1] (*Npm1*$^{+/TCTG}$) with constitutive *Flt3*-ITD (*Flt3*$^{+/ITD}$) mutant mice and used the Mx1Cre transgenic line to induce human *NPM1* mutation A expression in hematopoietic stem cells (HSC). Mice were treated through intra-peritoneal injections with a maximum tolerated dose of 5- azacytidine (5 mg/kg, every 3 days 5 doses) a demethylating agent.

Primary AML samples

Samples were collected from AML patients diagnosed at the Perugia University Hospital after informed consent. The protocol was approved by ethical committee of the University of Perugia (AIRC protocol 2016-04, ERC protocol 2017-09).

Peripheral blood counts

Mice were anesthetized with isoflurane followed by retro-orbital bleeding. Blood was taken into glass capillary tubes. Complete blood count was performed using an XE-2100 hematology automated analyzer (Dasit).

Flow Cytometry and Cell Sorting

Bone marrow cells were stained with the following antibodies from eBioscience: FITC-anti-Gr1, PE-anti-Mac1, APC-anti-cKit, FITC-anti-B220, PE-anti-CD3, FITC-anti-Ter119, PE-anti-CD41; biotin-conjugated antibodies against CD3, CD4, CD8, B220, Ter119, Mac-1, Gr-1, Sca-1, IL7R, CD5, phycoerythrin (PE)-Cy5–conjugated streptavidin, allophycocyanineFluor-780–conjugated

anti–c-Kit, FITC-conjugated anti-CD41, allophycocyanin-conjugated anti-FCgR, PE/CY7-conjugated anti-CD105 (BioLegend), and PE-conjugated anti-CD150 (BD Biosciences). Multiparameter flow cytometry was used to define and analyze the HSC compartment, including long-term hematopoietic stem cells (LT-HSC; lin-Sca-1+c-kit+ CD34−Flt3−), short-term HSC (ST-HSC; lin-Sca-1+c-kit+CD34+Flt3−) and multipotent progenitors (MPP; lin-Sca-1+c-kit+CD34+Flt3+). In addition, the myeloid progenitors' population was included and analyzed as common myeloid progenitors (CMPs Lin−Sca-1−cKit+CD34+FcγRII/IIIlo), granulocyte/monocyte progenitors (GMP Lin−Sca-1−cKit+CD34+FcγRII/IIIhi) and common megakaryocyte-erythroid progenitor (MEP Lin−Sca-1−cKit+CD34−FcγRII/IIIlo) populations. Cell acquisition and analysis were performed on a NAVIOS Beckman Coulter flow cytometer. Sorting experiments were performed using the FACSAriaIII cell sorter. Gates were drawn to exclude nonviable cells and debris. Part of the flow cytometry data was analyzed with FlowJo software (Tree Star, Ashland, OR).

In vitro hematopoietic colony-forming assay

For in vitro replating assays, a total of 105 BM cells were plated in duplicate in MethoCult M3434 (StemCell Technologies) containing rm SCF, rm IL-3, rh IL-6, rh EPO and grown at 37°C with 5% CO2. All colonies were quantified after 10-12 days of growth for detection of BFU-E, CFU-GM, CFU-GEMM in BM.

Cloning, Infection and Transplant

GATA1 mouse cDNA was cloned into the *pSico* vector[1], a gift from Tyler Jacks (Addgene plasmid #11578), containing loxP sites and GFP reporter protein allowing conditional *GATA1* expression.

HEK293T cell line was transfected with pSico/GATA1 vector with Lipofectamine and viral supernatant was used to infect Lin- BM cells. Infected cells were cultured in StemSpan serum-free medium add with 100 ng/ml mouse KIT ligand, 20 ng/ml human thrombopoietin, 100 ng/ml human

FLT ligand. Lethally irradiated mice were transplanted with infected cells in a range of $4x10^5$cells/mouse and sacrificed two months after transplantation for GATA1 re-expression and flow cytometry analysis.

Immunoblotting analysis

Gata1 protein was detected by WB analysis with an anti-rat primary antibody against GATA-1 (N6) clone sc-265 (Santa Cruz). Mouse a-tubulin antibody was used as control (Sigma).

qRT-PCR analysis

RNA was extracted with Trizol reagent and reverse-transcribed with PrimeScript RT Master Mix (Diatechlabline). Quantitative reverse transcriptase-PCR (qRT-PCR) was performed using SYBR Green Premix Ex Taq II (Diatechlabline) and a 7900 Fast Real-Time PCR System (Applied Biosystems, Foster City, CA). PCR amplifications were performed by incubation at 95°C for 10 minutes, followed by 40 cycles of incubations at 95°C for 15 seconds and 60°C for 1 minute. Data were analyzed according to the comparative Ct method and normalized to glyceraldehyde-3-phosphate dehydrogenase expression levels within each sample. The 2-DDCt method was used to calculate relative expression levels of target genes. Data analysis was performed using the SDS Enterprise Database (Applied Biosystems). The following primers were used to quantify *GATA1* mouse expression: GATA1-F 5'-CGTCATACCACTAAGGTGGCTGAAT-3'; GATA1-R 5'-GTGGAATCTGATGGTGAGGACA-3' in comparison with endogenous *GAPDH*: GAPDH-F 5'-TGTGTCCGTCGTGGATCTGA-3'; GAPDH-R 5'-CCTGCTTCACCACCTTCTTGA-3'. The following primers were used to quantify *GATA1* human expression: hGATA1F 5'-CTGTCCCCAATAGTGCTTATGG-3'; hGATA1R 5'-GAATAGGCTGCTGAATTGAGGG-3' in comparison with endogenous *GAPDH*: hGAPDH-F 5'-ATGGGGAAGGTGAAGGTCG-3'; hGAPDH-R 5'-GGGGTCATTGATGGCAACAATA-3'.

Methylation analysis by Bisulfite Treatment and qRT-PCR

We used the CpG Island searcher software to identify CpG islands within the mouse *GATA1* gene promoter region (http://urogene.org/cgi-bin/methprimer/methprimer.cgi/). Methylation analysis was performed using two different approaches following a common sodium bisulfite treatment of genomic DNA. In the first approach, sodium bisulfite–treated gDNA was PCR amplified, PCR products were cloned into the pCR2.1 TOPO vector (Invitrogen), and individual clones were sequenced. Primer sets amplified a 235-bp product located at the same genomic region of the mouse *Gata1* promoter (forward primers from -768 to -744 and reverse primers from -534 to -560). To explored the methylation status of the human GATA1 promoter we generated primer sets that amplified a 216 bp product within the described promoter region[2]. Gene-specific DNA methylation was determined with a OneStepqMethyl-Lite kit (Zymo Research, Irvine, CA, USA) and specific methyl primers and probes (PrimeTime qPCR Assay Integrated DNA technologies). Primers spanned a DNA region that is 350 bp and contains two MSRE sites. In the second approach, 20 ng of global DNA was incubated in the presence (test reaction) or absence (reference reaction) of methyl sensitive restriction enzymes (5 U each) at 37°C for 2 h, followed by real-time reverse transcription PCR (RTPCR) as described in the manufacturer's instructions. Percentage methylation was calculated using the formula 100x2-ΔCt , where ΔCt is the average Ct value from the test reaction minus the average Ct value from the reference reaction. Percentage methylation is relative to each experiment.

Gene expression profiling

Mouse gene expression profiles (GEPs) were generated using an Affimetrix probe MoGene-2_0-st-v1. Expression levels were processed using standard methods of normalization and significance analysis with R/Bioconductor[3,4]. The gene ontology analysis was performed in R/Bioconductor and represented with tree-map plots, including enrichment in "biological process", "cellular

component", "molecular function" obtained with genes with p-value < 0.05 and |fold change| < 1.5 as standard. Finally, the pathway analysis was done with R/Bioconductor querying KEGG 1, to select only the enriched pathways (p-value < 0.05 and |fold change| < 1.5) and Reactome 2 selecting the most targeted pathways, considering only differential expressed genes.

Accession Numbers

mRNA expression profiles data (GEO accession GSE16015) can be accessed in the Gene Expression Omnibus.

Statistical analysis

For survival studies, we used the method of Kaplan and Meier with a follow-up of 20 mice per genotype. For studies in which animals are sacrificed for analysis at different ages, we studied 4 mice per group per time point, and analyzed the data using analysis of variance. For *in vitro* assays, utilized a standard Students t-test. Statistical analysis was carried out on at least three independent experiments.

SUPPLEMENTARY REFERENCES

1. Ventura A, Meissner A, Dillon CP, McManus M, Sharp PA, Van Parijs L, Jaenisch R, Jacks T. Cre-lox-regulated conditional RNA interference from transgenes. Proc Natl Acad Sci U S A. 2004; 101(28):10380-5.

2. Nicolis S, Bertini C, Ronchi A, Crotta S, Lanfranco L, Moroni E, *et al.* An erythroid specific enhancer upstream to the gene encoding the cell-type specific transcription factor GATA-1. *Nucleic Acids Res* 1991 Oct 11; 19(19)**:** 5285-5291.

3. Kanehisa M, Goto S. KEGG: kyoto encyclopedia of genes and genomes. Nucleic Acids Res 2000;28(1):27-30.

4. Fabregat A, Sidiropoulos K, Garapati P, Gillespie M, Hausmann K, Haw R, *et al.* The Reactome pathway Knowledgebase. Nucleic Acids Res 2016;44(D1): D481-7

SUPPLEMENTARY FIGURE LEGEND

Figure S1. Phenotype of *Npm1/Flt3-ITD* leukemic mice.

(A) (i) Bone marrow cytospin (60 x magnification) from a representative healthy wild-type (WT) and a leukemic mouse displaying blasts infiltration. (ii) Immunohistochemistry staining of NPM1 in blasts showing aberrant expression of NPM1 in the cytoplasm (60 x magnification). (B) (i) Representative flow-cytometric analysis showing the gating of Gr1/Mac1, c-Kit+ and Gr1+/c-Kit+ cells in the BM of leukemic mice. (ii) Different percentage of immature and mature myeloid cells in the four different genotypes of the diseased mice. The more immature blasts (c-Kit+) were two-fold higher in *Npm1*$^{TCTG/TCTG}$;*Flt3*$^{+/ITD}$ (n=14), *Npm1*$^{+/TCTG}$;*Flt3*$^{ITD/ITD}$ (n=4) and *Npm1*$^{TCTG/TCTG}$;*Flt3*$^{ITD/ITD}$ (n=6) mice compared to *Npm1*$^{+/TCTG}$;*Flt3*$^{+/ITD}$ (n=4) (20.2±12.8%, 21.28±2.7% and 17.08±5.6% vs 10.7±7.5%, respectively). In *Npm1*$^{+/TCTG}$;*Flt3*$^{+/ITD}$ leukemic BM samples the percentage of Gr1+/c-Kit+ cells was lower than in other diseased genotypes (3.4±2.7% vs 8.6±4.4%, 7±2.2% and 7±2.6). More differentiated Gr1+/Mac1+ cells were reduced in double mutated BM samples that were *Flt3*-ITD homozygous compared to heterozygous, regardless the *NPM1* mutation allelic burden (29.9±3.5% and 30.8±7.2% vs 41.3±28.5% and 40.2±20%, respectively). (iii) c-Kit+/Gr1+Mac1+ cells ratio in the spleen of leukemic mice (n = 4 to 15 per genotype).

* p<0.05, ** p<0.01; unpaired t-test with Welch's correction.

Figure S2. Bone marrow B cell and B cell progenitors.

(A-C) Representative FACS plot (left panels) and relative frequency and number (right panels) of total B cell (A), PreproB and ProB (B), PreB, Immature and Recirculating (C) BM cells (n = 4 per group) in wild type and *Npm1*$^{\mathrm{TCTG/+}}$;*Flt3*$^{\mathrm{+/ITD}}$ mice.

** p<0.01, *** p<0.001; unpaired t-test with Welch's correction

Figure S3. Hematopoietic changes in pre-leukemic *Npm1*$^{+/TCTG}$;*Flt3*$^{+/ITD}$ mice.

(A) Significant differences in platelets number (PLT) in *Npm1*$^{+/TCTG}$;*Flt3*$^{+/ITD}$ preleukemic mice compared to *Npm1*$^{+/TCTG}$;*Flt3*$^{+/+}$, *Npm1*$^{+/+}$;*Flt3*$^{+/ITD}$ and *Npm1*$^{+/+}$;*Flt3*$^{+/+}$ littermate groups (n = 12 to 20 per genotype). (B) Significant higher spleen-to-body weight ratios in *Npm1*$^{+/TCTG}$;*Flt3*$^{+/ITD}$ mice compared with *Npm1*$^{+/TCTG}$, *Flt3*$^{+/ITD}$ and wild type littermate groups (0.017±0.012 vs 0.008±0.002, 0.005±0.0008, 0.005±0.001 respectively) (n = 4 to 15 per genotype). (C) (i-ii) Flow-cytometric analysis of immature erythroid and megakaryocytic compartments, including megakaryocyte progenitor (MkP), pre-CFU-E, CFU-E, proEry, pre-megakaryocyte-erythrocyte progenitors (PreMegE) and Ter119 population (n = 4 to 9 per genotype). (iii) The graph represents the mean number of the myeloid clonogenic progenitor cells obtained using clonogenic assays of three independent experiments.

* p<0.05, ** p<0.01, *** p<0.001; unpaired t-test with Welch's correction.

Figure S4. Transcriptional network analysis in total BM cells.

Red nodes represent products of upregulated genes and blue nodes represent products of downregulated genes in *Npm1*TCTG/TCTG;*Flt3*+/ITD leukemic mice compared to wild-type controls.

Figure S5 Transcriptional network analysis in lineage depleted bone marrow cells.

Red nodes represent products of upregulated genes and blue nodes represent products of downregulated genes in *Npm1*TCTG/TCTG;*Flt3*+/ITD lineage depleted BM cells from leukemic mice compared to wild-type controls.

Figure S6. The extent of GATA1 deregulation correlated with the degree of the myeloid phenotypic changes in mutant BM cells

(A) (i) *Npm1*$^{+/TCTG}$;*Flt3*$^{+/ITD}$ samples were binarized into low and high GATA-1 mRNA expression (n = 4) (ii) GATA1 protein expression in *Npm1*$^{+/TCTG}$;*Flt3*$^{+/ITD}$ samples relative to high and low mRNA expression (n = 4). (B) (i-iv) Significant differences in WBC counts, MPP and GMP compartments in the BM of *Npm1*$^{+/TCTG}$;*Flt3*$^{+/ITD}$ mice according to GATA1 mRNA expression levels. Percentage of GR1+Mac1+ cells in the spleen of *Npm1*$^{+/TCTG}$;*Flt3*$^{+/ITD}$ mice displaying either low or high GATA1 mRNA levels (n = 12 to 16 per genotype).

* p<0.05, ** p<0.01, ***p<0,001; unpaired t-test with Welch's correction

Figure S7. Cellular effects of enforced expression of GATA1 in pre-leukemic Npm1$^{+/TCTG}$;Flt3$^{+/ITD}$ cells

(A) An inducible GATA1 lentiviral system lead to GATA1 mRNA expression in the BM of mice transplanted with *Npm1*$^{+/TCTG}$;*Flt3*$^{+/ITD}$ LSK (n = 4 to 12). (B) (i) Representative FACS plot of hematopoietic stem cells (LT-HSC, ST-HSC and MPP) and progenitor populations (CMP, GMP, and MEP) in MOCK and GATA1 infected mice (n = 10 to 16). (ii) Frequency of MEP populations in MOCK and GATA1 infected mice. Values are derived from MOCK (n=10) and GATA1 (n=16) infected mice. Data represent the mean ± SD. (C) (i) Representative spleen size and average

spleen/body weights ratio (lower histogram) of MOCK (n=10) and GATA1 (n=16) infected mice. The data are presented as mean ± SD. (ii) Representative FACS plot of mature (Gr1-Mac1) and immature (Gr1-c-Kit) myeloid cells infiltrating the spleen of MOCK and GATA1 infected mice; (iii-iv) histograms showing average frequency of Gr1-Mac1 and Gr1-c-Kit cells according to the gating strategy on the facs plot.

* $p<0.05$, ** $p<0.01$; unpaired t-test with Welch's correction

Figure S8. Cellular and molecular changes in *Npm1*$^{+/TCTG}$*;Flt3*$^{+/ITD}$ mice treated with AZA

(A) Western blot analysis of GATA1 expression in *Npm1*$^{+/TCTG}$*;Flt3*$^{+/ITD}$ BM cells cultured in presence or absence of the proteasome inhibitor MG132. (B) GATA1 mRNA expression in the BM of AZA treated mice (n = 5). (C) (i-ii) Representative FACS plot of MEP and Ter119 cells in the BM of AZA treated mice (n = 5 to 12 per treatment group).

* $p<0.05$, ** $p<0.01$; unpaired t-test with Welch's correction

Figure S9. *GATA1* mRNA expression analysis of an independent AML database

(A) Analysis of GATA1 levels in AML samples including both normal and complex karyotype (n = 20 to 179 samples for each genetic group indicated in the figure). (B) Analysis of GATA1 levels in AML samples with normal karyotype (NK) only.

* $p<0.05$ comparing all the groups; one-way ANOVA analysis

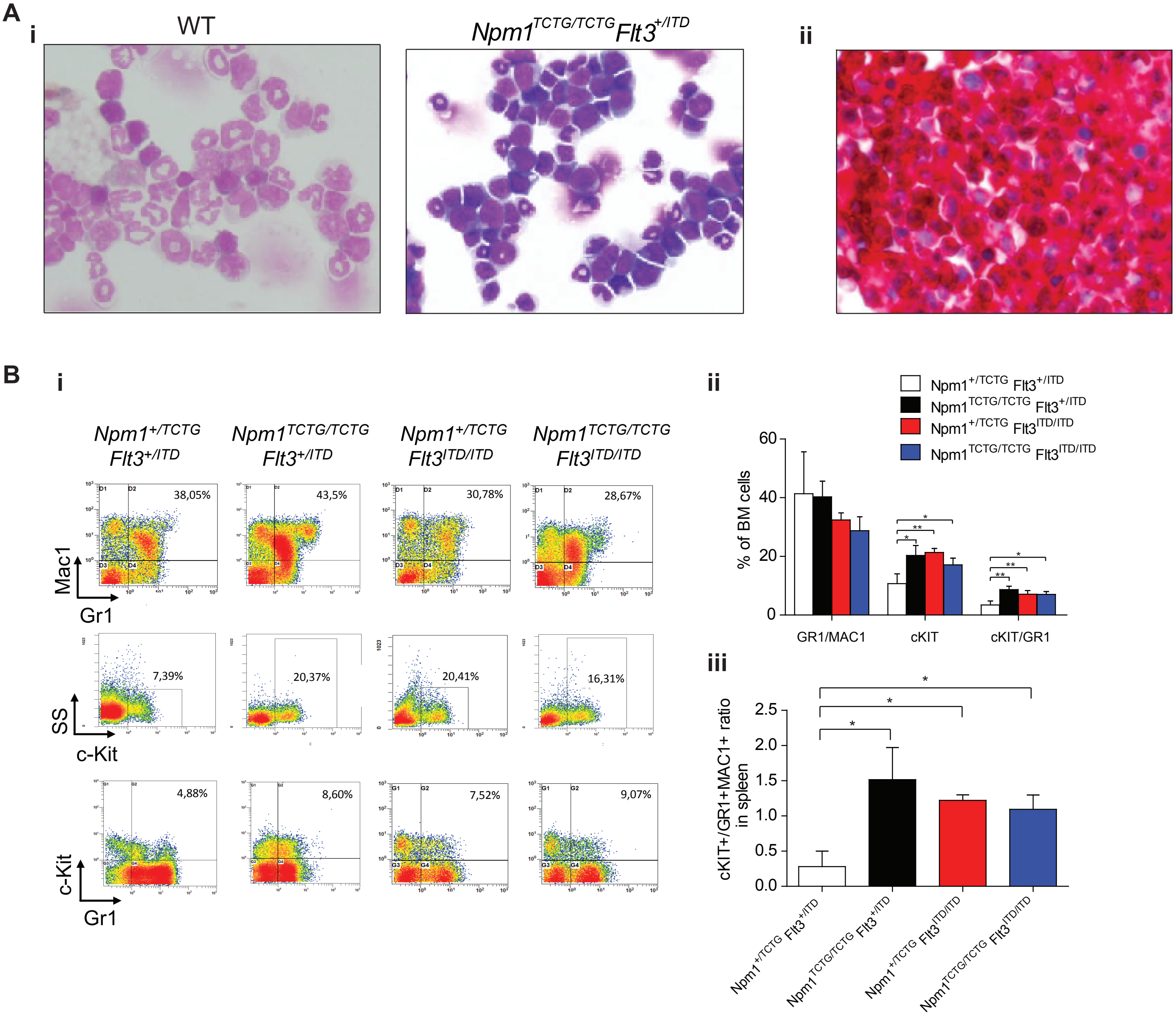

Figure S1: Sportoletti et al.

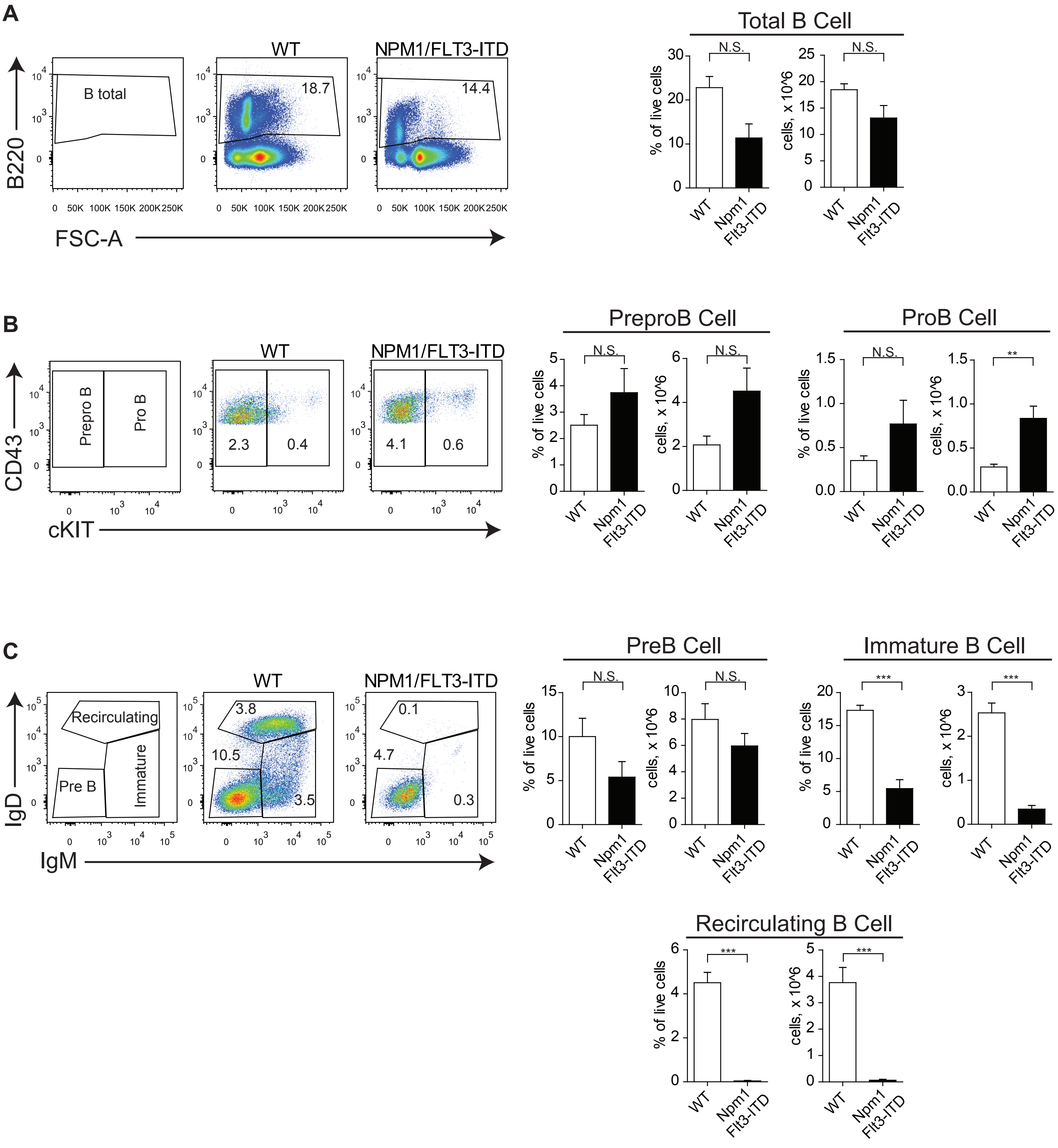

Figure S2: Sportoletti et al.

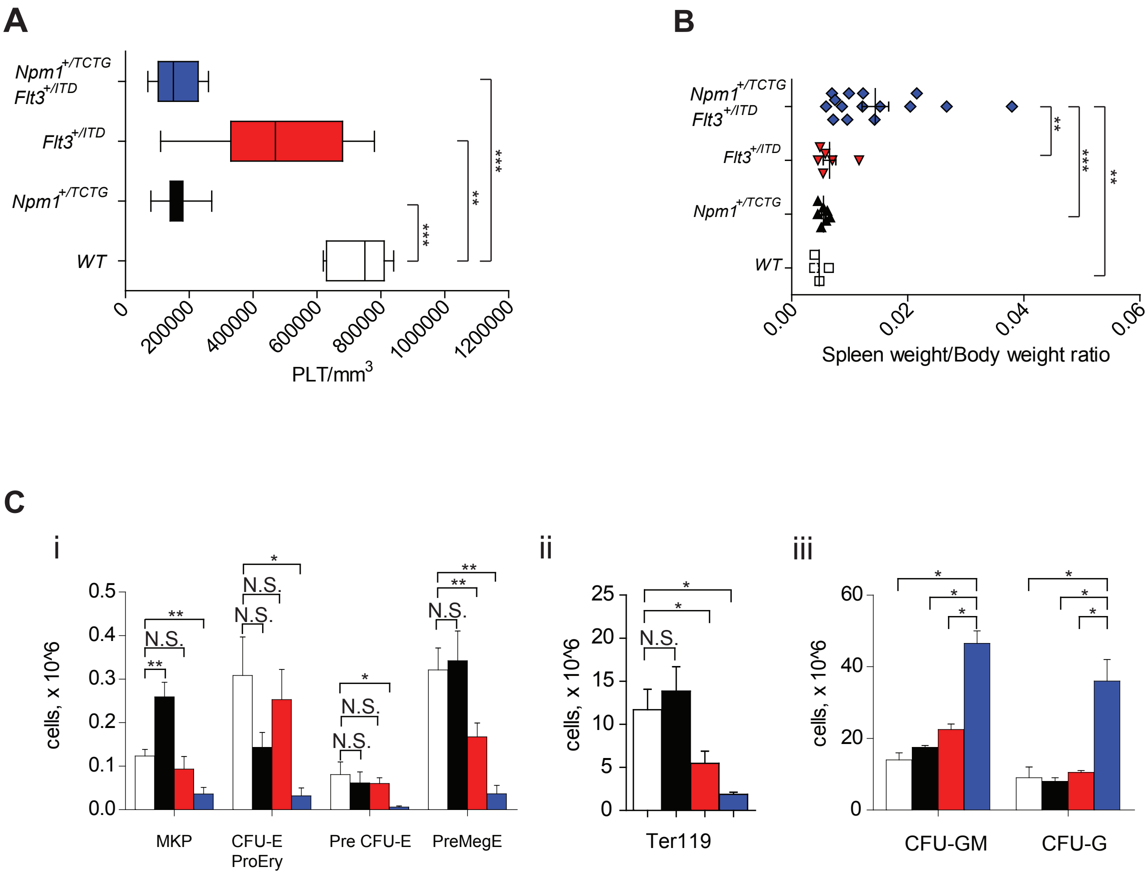

Figure S3: Sportoletti et al.

Factors involved in megakaryocyte development and platelet production

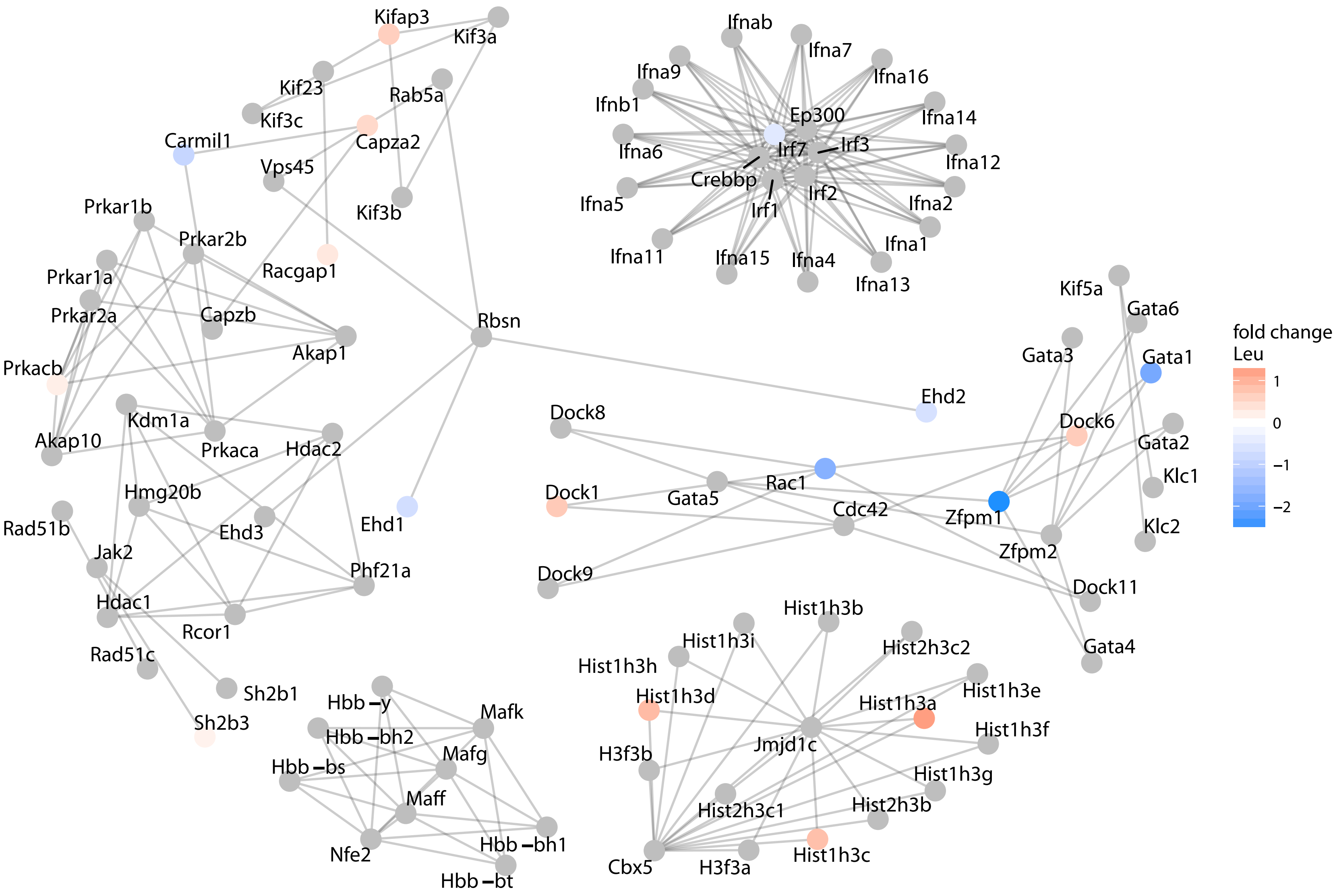

Figure S4: Sportoletti et al.

Factors involved in Megacayocyte and platelet production

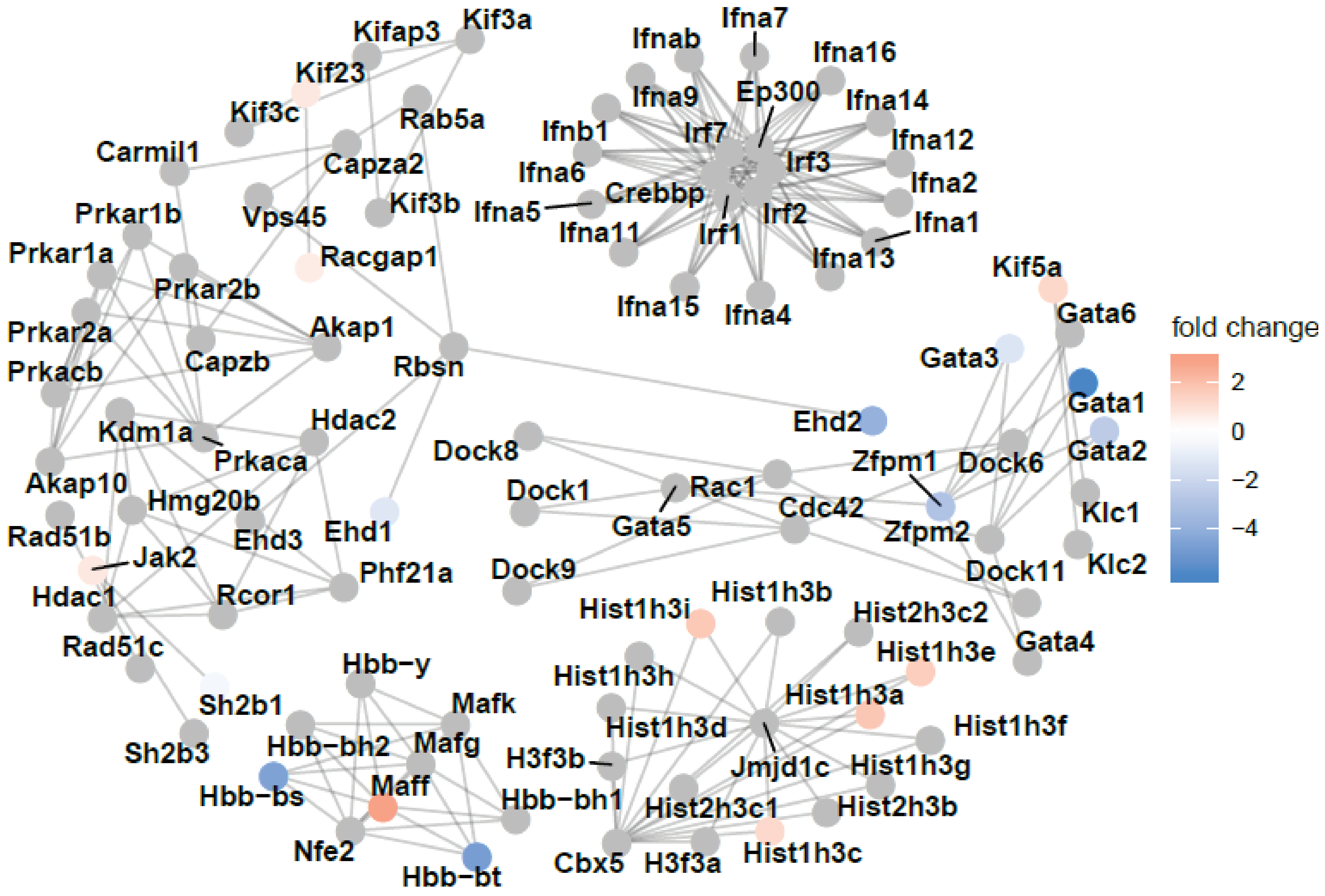

Figure S5: Sportoletti et al.

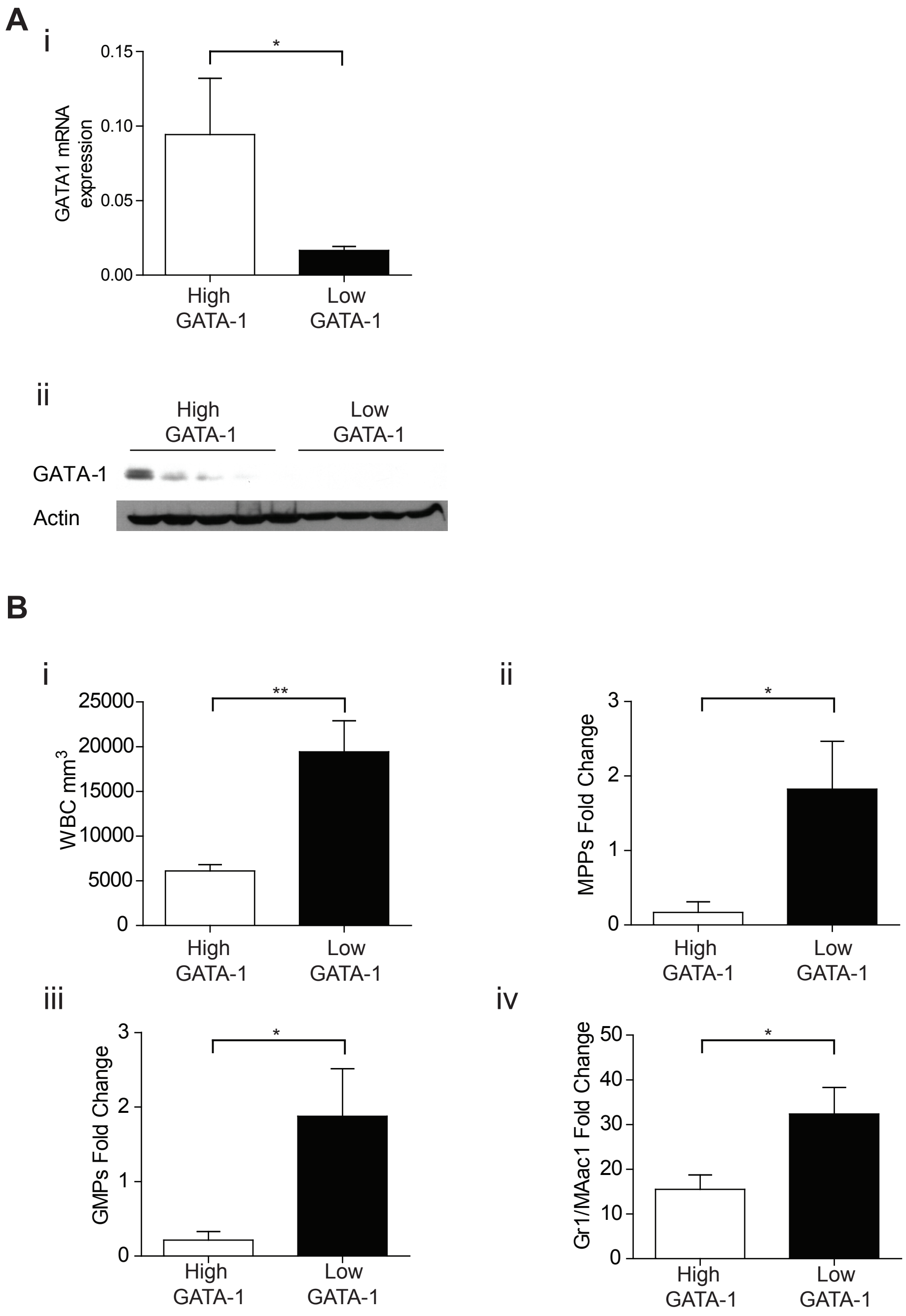

Figure S6: Sportoletti et al.

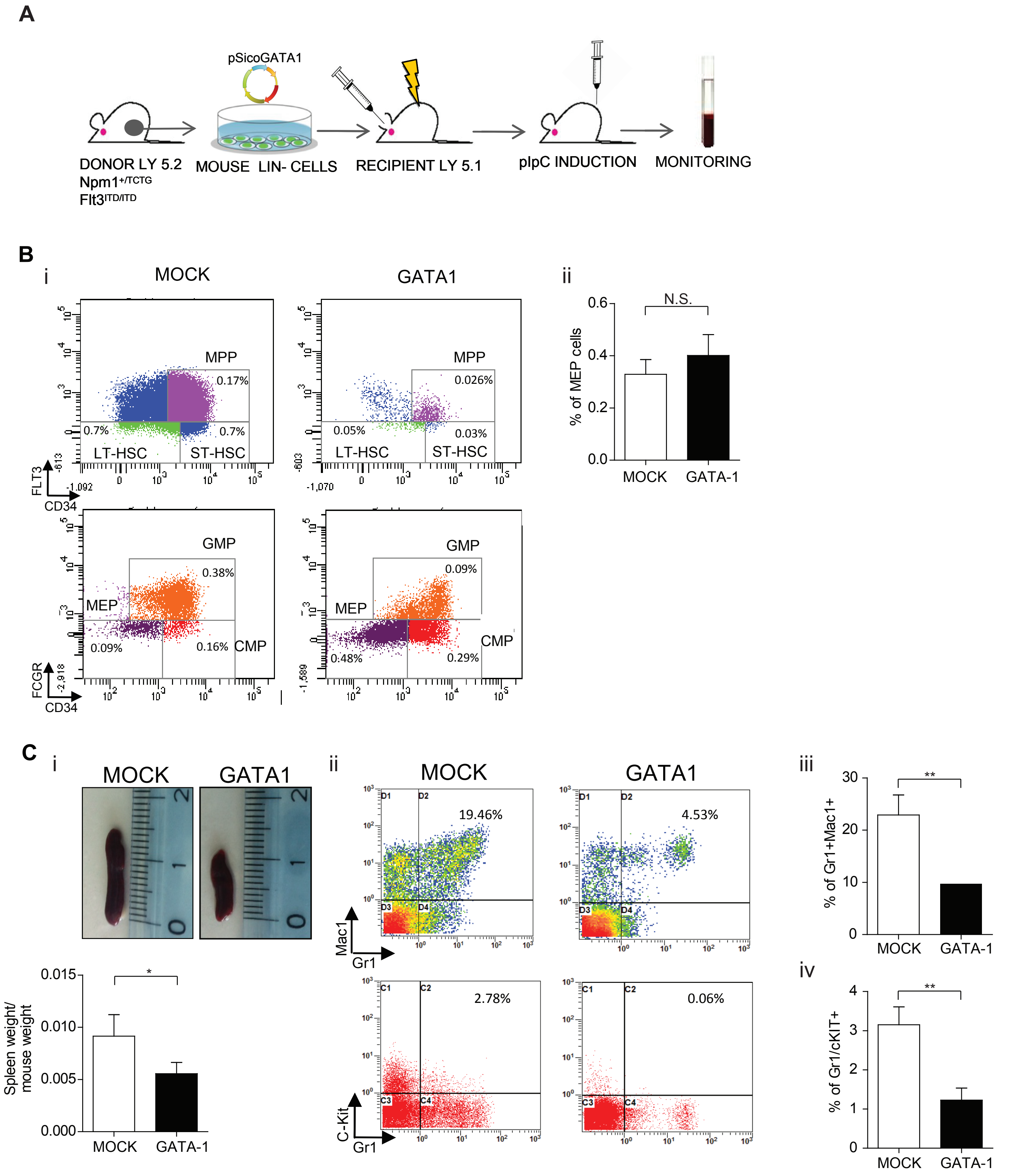

Figure S7: Sportoletti et al.

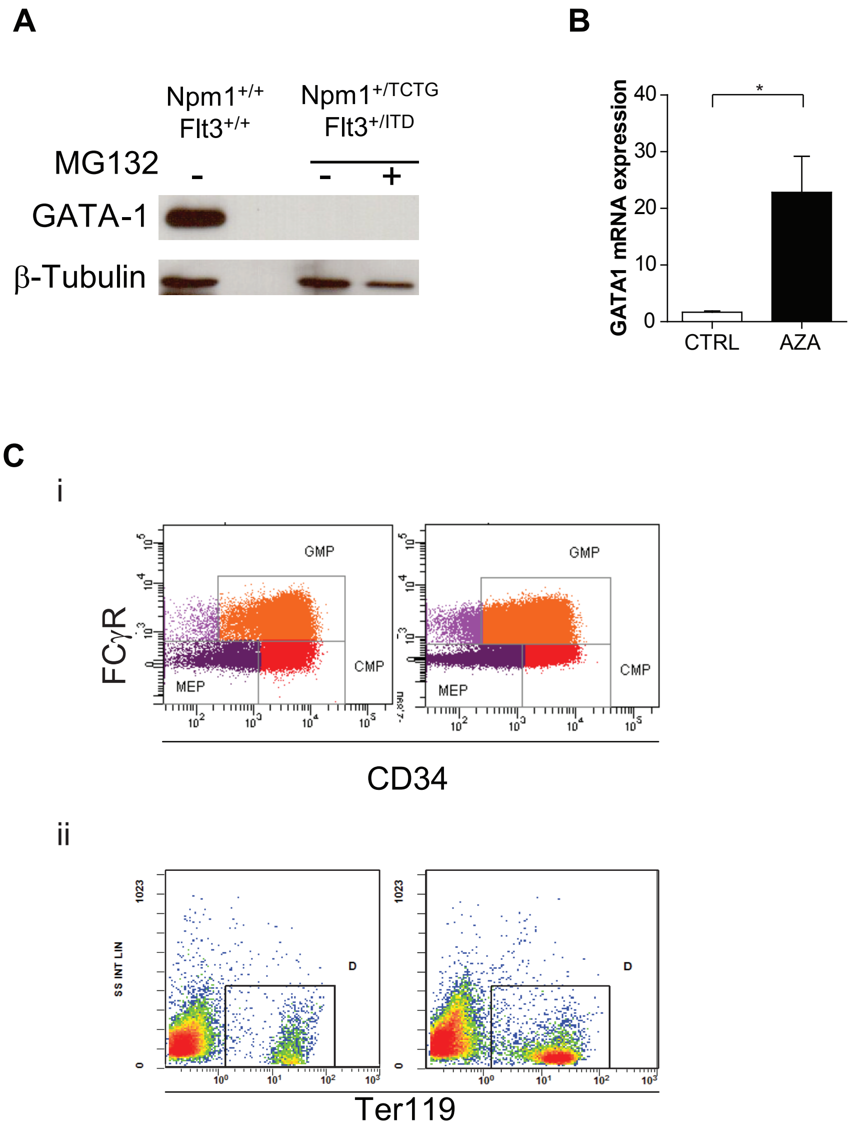

Figure S8: Sportoletti et al.

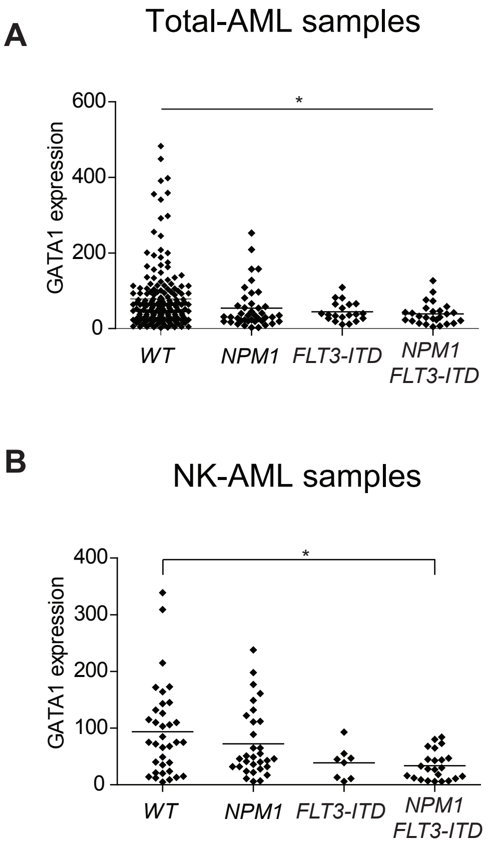

Figure S9: Sportoletti et al.

Npm1$^{TCTG/TCTG}$**;*Flt3***$^{+/ITD}$				***Npm1***$^{TCTG/TCTG}$				***Flt3***$^{+/ITD}$			
**UP**	**values**	**DOWN**	**values**	**UP**	**values**	**DOWN**	**values**	**UP**	**values**	**DOWN**	**values**
Plxdc2	918.514	Cd19	-993.097	Apol11a	267.269	Ighv1-12	-328.212	Ifi205	431.731	Trim12a	-544.978
Cp	743.385	Spib	-967.337	Apol11b	237.619	Oas1g	-301.488	Cd209a	283.865	Npy	-299.044
Tmem178	711.948	Rhag	-958.368	Ddx3y	180.541	Igh-VJ558	-286.625	Fosb	158.444	Gm1966	-137.805
Nov	566.504	Ly6d	-932.738	Cyp2b10	114.246	Xist	-202.997	Socs2	125.124	Hspa1a	-129.036
BC064078	542.465	Ighv1-12	-904.936	Stfa3	101.652	Igkv8-30	-131.275			Mir144	-126.438
Adgrg5	438.509	Ighg3	-864.241	Uty	81.545					Erdr1	-107.856
Hoxa9	415.979	Kel	-856.067							Egr1	-105.041
Ifi205	375.145	Il7r	-834.324								
Vcan	373.117	Igll1	-828.473								
Olfm1	361.571	Nxpe2	-827.136								
Fabp5	361.004	Redrum	-804.044								
Gpnmb	354.033	Car2	-799.962								
Kcnq3	343.887	Ctse	-781.429								
Il6st	341.701	Pou2af1	-777.752								
Shtn1	341.311	Ikzf3	-772.414								
Eif2s3y	341.153	Dntt	-760.881								
Rasgrf2	333.348	Pklr	-745.948								
Itga1	328.264	Blnk	-741.339								
Clec4b1	327.084	Abcb4	-738.785								
S100a4	322.329	A730089K16Rik	-729.431								
Rnase2a	321.661	Klf1	-726.388								
Sulf2	321.172	Trim12a	-690.943								
I730030J21Rik	320.019	Ank1	-690.755								
Bex6	316.366	Cldn13	-683.573								
Igfbp7	311.822	Slc38a5	-680.962								
Cd209a	308.543	Akap12	-672.877								
Wfdc17	302.309	Vpreb3	-641.807								
Nrg1	299.002	Mt2	-636.524								
Cfh	294.193	Mboat2	-631.716								
Klrb1f	293.093	Spta1	-618.918								
Qpct	293.091	Igkv6-23	-617.533								
Ccr2	292.922	Cd72	-615.588								
Pid1	288.687	Aqp1	-611.981								

Enah	284.979	Rhd	-608.965								
Dusp22	283.811	Cmah	-607.644								
Tmem176a	282.973	Igk-V8	-603.192								
Kdm5d	103.697	Gypa	-587.857								
Abcb1b	34.169	Fcer2a	-583.047								
Cd34	30.799	Oas1g	-580.198								
Cd209c	28.731	Zfpm1	-579.963								
Uty	16.437	Cd22	-573.019								
		Tspan33	-569.515								
		Paqr9	-564.136								
		Gfi1b	-563.572								
		Hemgn	-555.876								
		S1pr1	-545.576								
		Blk	-540.033								
		Sptb	-535.097								
		Slamf6	-533.604								
		Btnl10	-529.937								
		Cr2	-518.154								
		Igkv15-103	-514.258								
		Igkv14-126	-507.943								
		Tmem56	-505.151								
		Add2	-496.229								
		Blvrb	-492.371								
		Cd79b	-491.904								
		Cyp2r1	-487.641								
		Igkv1-133	-485.448								
		Fcrla	-484.977								
		Igkv6-14	-471.498								
		Ighv1-61	-467.061								
		Cpox	-465.465								
		Igh-V11	-462.097								
		Ahsp	-457.493								
		Asprv1	-457.493								
		Ighv1-62	-454.098								
		Igk-V28	-449.261								
		Atp1b2	-445.944								
		Atp1b1	-444.398								

		Trib2	-430.362								
		Bach2	-429.619								
		Chst3	-427.617								
		Ighv1-54	-425.035								
		Gdpd1	-424.958								
		Mfsd2b	-423.806								
		Cdc42bpa	-416.666								
		Tspo2	-416.645								
		Epb42	-415.387								
		Epor	-415.143								
		Aldh1a7	-411.238								
		Slc22a23	-405.529								
		Spire1	-402.132								
		5430401H09Rik	-400.369								
		Myo1d	-397.792								
		Slc43a1	-394.622								
		D13Ertd608e	-394.302								
		Igkv1-135	-391.675								
		Ighv1-19	-389.262								
		Fam129c	-386.723								
		Tmem120b	-386.333								
		Slc4a1	-383.228								
		Gata1	-378.518								
		Lef1	-376.188								
		Igkv9-120	-373.911								
		Ralgps2	-373.095								
		Slc40a1	-372.447								
		Ighm	-371.914								
		Nfia	-371.534								
		Myh10	-370.861								
		Cd2	-366.438								
		Igh-VJ558	-365.404								
		Gzma	-362.403								
		Chchd10	-362.285								
		Cd55	-361.105								
		Alad	-360.122								
		Trim30d	-359.984								

		Bank1	-359.755								
		Trim10	-359.188								
		Chil5	-357.862								
		Igkv1-117	-353.472								
		Igkv8-30	-353.127								
		Gdf3	-352.374								
		Il1f9	-351.122								
		Slc6a20a	-350.745								
		Igkv4-70	-350.324								
		Tcrb-J	-348.952								
		Ighv10-3	-345.699								
		Il1rl1	-345.313								
		Gimap6	-344.146								
		Tom1l1	-341.951								
		Dennd5b	-341.618								
		Tfr2	-337.752								
		Gstm5	-337.505								
		Ighv1-53	-337.236								
		Iglv1	-336.275								
		Sec14l2	-335.892								
		Crip2	-333.482								
		Igkv9-129	-333.179								
		Rag2	-333.168								
		Gpr174	-332.829								
		Igkv4-72	-332.716								
		Sox6	-332.069								
		Iglv2	-326.119								
		Hmbs	-325.256								
		Agtr1a	-319.422								
		Npy	-319.108								
		Rasgrp3	-313.834								
		Ammecr1	-312.093								
		Ntn4	-311.162								
		Ighv9-4	-309.807								
		Chil1	-308.504								
		Serinc5	-308.482								
		Plpp1	-303.489								

		Gbp8	-303.091								
		Igk-V4	-301.601								
		Gpc4	-301.236								
		Igkv4-55	-298.149								
		Cd300ld	-298.083								
		Ly6g	-297.405								
		Gbp4	-296.996								
		Stard10	-296.342								
		Sdc4	-295.951								
		Tspan13	-295.166								
		Gfra2	-294.434								
		Igkv8-19	-294.068								
		Igh-VX24	-291.912								
		Fhdc1	-290.883								
		Cyth3	-290.774								
		Igh-V7183	-290.682								
		Cd59a	-289.759								
		St6gal1	-288.029								
		Ighv6-6	-286.839								
		Xrcc5	-285.957								
		Bex4	-283.822								
		Egr1	-264.866								
		St3gal6	-243.159								
		Cd79a	-242.786								
		Klk1	-239.883								
		Ms4a1	-225.907								
		Siglech	-222.076								
		LOC102642252	-218.101								
		Ifi214	-215.567								
		Igk	-214.573								
		Pla2g4c	-212.637								
		Mir144	-211.178								
		Ebf1	-202.502								
		Cecr2	-186.327								
		Pax5	-151.839								
		Slc25a21	-149.487								
		Fcmr	-141.977								

		Ermap	-139.845								
		Car1	-139.336								
		Ces2g	-121.883								
		Pkhd1l1	-120.679								
		Rac1	-113.331								
		Scd1	-110.097								
		Gm15915	-109.289								
		Xist	-109.029								
		Igkv4-53	-104.692								
		Gm32819	-94.442								
		Aldh1a1	-75.918								
		Tspan8	-70.562								
		Cpm	-64.355								
		Reep6	-54.164								
		Cacna1e	-52.544								
		Cd38	-51.526								
		Cd5l	-39.083								
		BE692007	-36.643								
		Zdhhc14	36.523								
		Asb17os	-33.787								
		Mns1	-32.348								
		Atp7b	-29.788								
		Igkv3-4	-29.137								
		Traf4	-28.918								
		Abcg4	-26.861								
		Aff2	-34.532								
		Nckap1	-28.961								

Table S1. List of differentially expressed genes and relative values in the bone marrow of *Npm1*$^{TCTG/TCTG}$;*Flt3*$^{+/ITD}$; *Npm1*$^{TCTG/TCTG}$;*Flt3*$^{+/+}$ and *Flt3*$^{+/ITD}$, compared to *Npm1*$^{+/+}$;*Flt3*$^{+/+}$ Wild type control.

$Npm1^{TCTG/TCTG};Flt3^{+/ITD}$			
**DOWN**	**values**	**UP**	**values**
Gdf3	-886,604	Nov	353,3368
Blk	-873,292	Adgrg5	21,38727
Slc4a1	-285,161	Plxdc2	12,90118
Aldh1a1	-282,301	Cp	12,19327
Tspan8	-275,819	Igfbp7	10,89965
Igkv9-129	-230,698	Tmem178	8,850796
Btnl10	-217,773	Cd209c	7,352113
Paqr9	-212,663	Kcnq3	6,871774
Cd19	-206,606	Enah	6,716575
Trim10	-201,191	BC064078	5,372653
Epor	-163,395	Cd209a	4,694917
Rhd	-158,456	Rasgrf2	4,509685
Sptb	-150,067	Ifi205	3,803583
Add2	-145,859	Clec4b1	3,668178
Trib2	-143,066	S100a4	3,162371
Tspo2	-136,672	Sulf2	2,724305
A730089K16Rik	-132,47	Wfdc17	2,663991
Igkv15-103	-120,003	Qpct	2,494077
Ermap	-119,087	Pid1	2,489407
Ank1	-116,077	Dusp22	2,431463
Igkv4-55	-111,959	Klrb1f	2,427726
Gypa	-103,248	Egr1	2,422851
Spta1	-101,581	Itga1	2,407483
Gpc4	-99,7659	Olfm1	2,160244
Pkhd1l1	-95,0268	Klk1	2,102996
Rhag	-91,162	Abcb1b	2,099031
Redrum	-90,2244	Fabp5	2,087197
Cldn13	-88,929	Hoxa9	2,075765
Asb17os	-87,9272	Shtn1	1,914062
Igkv1-133	-86,3035	Ccr2	1,704657
Pax5	-78,4597	Il6st	1,659123
Car1	-78,4097	Nrg1	1,599928
Slc38a5	-76,9679	Bach2	1,595562
Igkv9-120	-70,4622	Rac1	1,428006
Igkv3-4	-67,8365	Bex6	1,416588
Ms4a1	-67,5753	Cfh	1,341099
Slc25a21	-66,0792	Tmem176a	1,2806
Agtr1a	-64,1694	Uty	1,21077
Pklr	-62,0641	Eif2s3y	1,146226
Gata1	-61,9127		
Kel	-58,0389		
Tmem56	-56,4067		
Klf1	-55,0319		

Sec14l2	-52,1195		
Aldh1a7	-50,3731		
Cr2	-47,5868		
Ces2g	-44,0827		
Aqp1	-43,3569		
Abcg4	-41,9547		
Sox6	-41,0053		
Ighv1-53	-37,8551		
Fcmr	-37,5665		
Igkv4-70	-37,5207		
Bex4	-35,0058		
Mfsd2b	-34,7841		
Ighg3	-34,7053		
Ebf1	-33,3863		
Cd5l	-30,6045		
Igkv6-14	-28,8325		
Cd2	-28,554		
Hemgn	-27,4618		
Cecr2	-27,1872		
Gm15915	-26,9538		
Iglv1	-25,9822		
Ighv1-12	-25,8761		
Nxpe2	-23,7896		
Igkv4-72	-22,4887		
Cd79a	-21,6583		
Pou2af1	-21,6428		
Ntn4	-20,6278		
Aff2	-20,5824		
Vpreb3	-20,472		
Igkv8-30	-20,3078		
Slc6a20a	-20,2492		
Myo1d	-19,8785		
Ighv1-62	-17,9152		
Ighv1-61	-17,843		
Ikzf3	-17,6947		
Asprv1	-16,8825		
S1pr1	-16,7871		
Mt2	-16,5731		
Cyp2r1	-15,9097		
Cpm	-15,7081		
Car2	-14,8477		
Ighv6-6	-14,5382		
Ighm	-14,2451		
Igkv4-53	-13,3716		
Fhdc1	-13,2208		
Fcrla	-12,9398		

Il7r	-12,6937		
Igkv1-135	-12,319		
Igkv8-19	-11,8922		
Ctse	-11,8033		
Oas1g	-11,4272		
Gpr174	-11,3469		
Cmah	-10,944		
Chst3	-10,8013		
Lef1	-10,7708		
Ighv1-54	-10,5236		
Ly6d	-10,4556		
Ighv10-3	-10,4487		
Iglv2	-9,7884		
Igkv6-23	-9,2602		
Myh10	-9,08603		
Dennd5b	-8,9803		
Sdc4	-8,51107		
Gdpd1	-8,44204		
Ighv1-19	-8,33283		
Blnk	-8,22205		
Dntt	-8,10243		
St3gal6	-8,03111		
Atp7b	-7,88364		
St6gal1	-7,779		
Zfpm1	-7,70573		
Cd38	-7,05817		
Igkv1-117	-6,95727		
Slc40a1	-6,38227		
Gbp4	-6,35128		
Igkv14-126	-6,10648		
Akap12	-5,95145		
Cd55	-5,83077		
Rag2	-5,57938		
Abcb4	-5,47232		
Bank1	-5,35467		
Atp1b2	-5,30655		
Chil5	-4,93554		
Tom1l1	-4,89053		
Il1f9	-4,85467		
Cacna1e	-4,68883		
Xrcc5	-4,66601		
Cd59a	-4,61453		
Blvrb	-4,53239		
Cd300ld	-4,49322		
Ighv9-4	-4,39605		
Siglech	-4,315		

Tmem120b	-4,18559		
Gfi1b	-4,17193		
Tfr2	-4,07557		
BE692007	-4,05131		
Atp1b1	-3,67232		
Reep6	-3,5998		
Slc43a1	-3,52854		
Gstm5	-3,5126		
Chil1	-3,50414		
Ly6g	-3,40398		
Cpox	-3,28971		
Igll1	-3,21557		
Gimap6	-3,14347		
Gbp8	-3,133		
Nckap1	-3,1036		
Slamf6	-2,9528		
Pla2g4c	-2,94508		
Ralgps2	-2,72054		
Cdc42bpa	-2,7131		
Cd72	-2,65915		
Alad	-2,62918		
Gzma	-2,58262		
Hmbs	-2,48448		
Gfra2	-2,41796		
Tspan33	-2,28007		
Vcan	-2,15794		
Stard10	-2,15592		
Rasgrp3	-1,99385		
Gpnmb	-1,97158		
Nfia	-1,96779		
Cd22	-1,92221		
Trim30d	-1,92031		
Chchd10	-1,88921		
Spib	-1,88421		
Traf4	-1,84977		
Ammecr1	-1,84632		
Cd79b	-1,82618		
Tspan13	-1,71759		
Zdhhc14	-1,69435		
Slc22a23	-1,68204		
Crip2	-1,68132		
Spire1	-1,67791		
Fcer2a	-1,62478		
Serinc5	-1,57831		
Il1rl1	-1,49619		
Mboat2	-1,49484		

D13Ertd608e	-1,49095		
Scd1	-1,40765		
Cd34	-1,39916		
Cyth3	-1,37303		
Mns1	-1,33454		
Rnase2a	-1,32368		
Fam129c	-1,24192		
Trim12a	-1,20898		
Xist	-1,05052		
Kdm5d	-1,03279		

Table S2. List of differentially expressed genes and relative values in the Lineage negative bone marrow cells of *Npm1*$^{TCTG/TCTG}$;*Flt3*$^{+/ITD}$; compared to Wild type control.
